# Apparent Half-Lives of Dioxins, Furans, and Polychlorinated Biphenyls as a Function of Age, Body Fat, Smoking Status, and Breast-Feeding

**DOI:** 10.1289/ehp.11781

**Published:** 2008-10-03

**Authors:** Meghan O’Grady Milbrath, Yvan Wenger, Chiung-Wen Chang, Claude Emond, David Garabrant, Brenda W. Gillespie, Olivier Jolliet

**Affiliations:** 1 Department of Environmental Health Sciences, University of Michigan School of Public Health, Ann Arbor, Michigan, USA;; 2 Department of Environmental and Occupational Health, Faculty of Medicine, University of Montreal, Montreal, Quebec, Canada;; 3 Department of Biostatistics, University of Michigan School of Public Health, Ann Arbor, Michigan, USA

**Keywords:** elimination rate, half-life, pharmacokinetics, polychlorinated biphenyls, poly-chlorinated dibenzofurans, polychlorinated dibenzo-*p*-dioxins

## Abstract

**Objective:**

In this study we reviewed the half-life data in the literature for the 29 dioxin, furan, and polychlorinated biphenyl congeners named in the World Health Organization toxic equivalency factor scheme, with the aim of providing a reference value for the half-life of each congener in the human body and a method of half-life estimation that accounts for an individual’s personal characteristics.

**Data sources and extraction:**

We compared data from > 30 studies containing congener-specific elimination rates. Half-life data were extracted and compiled into a summary table. We then created a subset of these data based on defined exclusionary criteria.

**Data synthesis:**

We defined values for each congener that approximate the half-life in an infant and in an adult. A linear interpolation of these values was used to examine the relationship between half-life and age, percent body fat, and absolute body fat. We developed predictive equations based on these relationships and adjustments for individual characteristics.

**Conclusions:**

The half-life of dioxins in the body can be predicted using a linear relationship with age adjusted for body fat, smoking, and breast-feeding. Data suggest an alternative method based on a linear relationship between half-life and total body fat, but this approach requires further testing and validation with individual measurements.

Polychlorinated dibenzo-*p*-dioxins (PCDDs), dibenzofurans (PCDFs), and biphenyls (PCBs) are lipophilic and can persist in the body for years (Schecter et al. 2006). An individual’s body burden is a product of multiple years of exposure (Pinsky and Lorber 1998) and a lifetime of varying elimination rates. Different congeners of PCDDs, PCDFs, and PCBs each have different persistence in the human body, reflected in their different reported half-lives. The apparent half-life, defined as the change in concentration in the body over time, is the net result of elimination from the body, changes in body composition, and intake from the environment. For each congener, variation in half-life exists both among individuals and within the same individual over his or her lifetime. This variability can be partially attributed to personal characteristics, including age, body fat, smoking status, and breast-feeding. The factors that affect elimination rates must be taken into account when predicting past exposures and body burdens of these chemicals and when comparing current serum congener profiles to exposure media.

## Age

In a study of German chemical workers, half-lives of numerous dioxins and furans were positively associated with increasing age (Flesch-Janys et al. 1996). This is consistent with a study on the Yusho and Yu-Cheng cohorts of half-lives of penta- (Pe), hexa- (Hx), and hepta (Hp) CDFs (Leung et al. 2007). Studies on the Ranch Hand cohort show a slight negative association (Wolfe et al. 1994) or no association (Michalek et al. 1996), but this may be due to the narrow age range characterizing these cohorts. Studies with child or infant subjects report significantly shorter half-lives than do studies with adult cohorts (Kreuzer et al. 1997; Leung et al. 2006, 2007). In children < 18 years of age exposed during the incident in Seveso, Italy, a strong association between half-life and age was found, and children had significantly shorter half-lives than did adults (Kerger et al. 2006).

The rapid growth of neonates and children, especially in lipid stores, can result in a dramatically reduced apparent half-life through dilution (Clewell et al. 2004). However, the effect of dilution alone is not sufficient to create the observed reduction in apparent half-life; it may also be due to a faster metabolism, an increased rate of fecal lipid excretion, or a combination of these events (Abraham et al. 1996; Kerger et al. 2007b). As children age, their rate of growth slows, and the effect of elimination on apparent half-life becomes more important than that of dilution.

The relationship between age and half-life is complex because age is strongly associated with other factors that affect half-life length (e.g., smoking status, percent body fat). As humans age, they generally experience an increase in and a redistribution of body fat as well as a relative change in organ sizes, causing a redistribution of lipophilic chemicals that greatly alters their rates of elimination (Van der Molen et al. 1996). Additionally, age may have an independent effect through an age-related reduction in hepatic elimination capacity (Aylward et al. 2005). A strong cohort effect is seen in cross-sectional studies, caused by varying levels of persistent chemicals in the environment. During the 1960s and 1970s, environmental levels of dioxins were much higher than they are today, leading to higher body burdens of the more persistent congeners in older people, above the level expected from persistence alone (Pinsky and Lorber 1998).

## Smoking status

Smoking has been associated with lower levels of dioxins and dioxin-like compounds. Active smokers have lower PCDD, PCDF, and PCB serum levels than do both nonsmokers and passive smokers (Brown and Lawton 2001; Chen et al. 2005), and levels of dioxin-like PCBs in human milk are negatively related to the smoking habits of the mothers (Uehara et al. 2007). This is in agreement with results of Flesch-Janys et al. (1996), who observed that the half-lives of some PCDD and PCDF congeners appeared to be dependent on smoking status. They observed a significantly faster decay in smokers, with increases ranging from 30% [2,3,7,8-tetrachlorodibenzo-*p*-dioxin (TCDD)] to 100% 1,2,3,4,7,8-HxCDD. Smoking induces the transcription of cytochrome P450 (CYP) 1A2 and other enzymes responsible for the elimination of dioxin and dioxin-like compounds, most likely through the activation of the aryl hydrocarbon receptor by polycyclic aromatic hydrocarbons in tobacco smoke (Zevin and Benowitz 1999). The total effect of smoking on half-life may be through this increased induction of dioxin-degrading enzymes, or through a combination of other physiologic effects.

## Body burden

Dioxins are known to up-regulate the enzymes responsible for their own elimination. Modeled and experimental data in rats show that at high exposures the induction of CYP1A2 is a more important factor for persistence in the body than are differences in adipose tissue distribution (Emond et al. 2006). A concentration-dependent biphasic elimination rate has been identified in cases of acute poisoning (Abraham et al. 2002), in the Seveso incident (Aylward et al. 2005; Michalek et al. 2002), in children (Kerger et al. 2006), and in the Yusho and Yu-Cheng rice oil poisonings (Leung et al. 2007; Ryan et al. 1993). Human data suggest that the serum concentration where this transition occurs is 700 ppt (Kerger et al. 2006) for TCDD and 1,000–3,000 ppt for PCDFs (Leung et al. 2005). These concentrations are considerably higher than those measured in people exposed to present background levels.

## Body fat

Because dioxins, furans, and PCBs are highly lipophilic, they partition preferentially in adipose tissue and other body fat. High amounts of adipose tissue, estimated by body mass index [BMI; weight (kilograms)/height^2^ (meters)], are associated with higher serum levels of dioxins and furans (Collins et al. 2007). Because adipose tissue acts as a reservoir for these chemicals, increases in adipose tissue result in their storage rather than transportation to excretory and metabolizing organs. Models based on the rat data demonstrate a linear relationship between increasing fat mass and half-life length at low body burdens, with the impact of adipose tissue on half-life becoming less important at high body burdens (Emond et al. 2006).

The relationship between percent body fat and half-life is apparent throughout the Ranch Hand study (Michalek et al. 1992, 1996; Michalek and Tripathi 1999), but these studies did not find a significant relationship between half-life and short-term changes in percent body fat. These findings are supported by the German occupational cohort, where a 1% increase in percent body fat was associated with a decay rate decrease in the range of 0.0031 ng/kg/year (1,2,3,6,7,8-HxCDD) to 0.0063 ng/kg/year (1,2,3,4,6,7,8-HpCDD) for dioxins, and about 0.005 ng/kg/year for furans (Flesch-Janys et al. 1996). This study did show an increased decay rate in workers with intermediate weight loss, but in a limited number of people (*n* = 3). Half-life is moderately correlated with both BMI and body fat mass in children, but longitudinal data from children are difficult to interpret because of their fast growth and simultaneous age-related changes (Kerger et al. 2006).

## Breast-feeding

For women, lactation can be the major route of elimination of many persistent lipophilic chemicals (Abraham et al. 1996; Schecter et al. 1996). Twenty percent or more of the maternal body burden of some persistent pollutants, such as PCBs, can be transferred during 6 months of lactation (Landrigan et al. 2002; Niessen et al. 1984). The reduction of half-life due to breast-feeding is both congener specific and duration dependent. The amount of fat in breast milk varies over time, affecting the partitioning of chemicals from the body (Clewell and Gearhart 2002). Different congeners partition differently into the breast milk from the blood (Schecter et al. 1996, 1998), and this distribution is thought to be dependent on the molecular weight of the congener. Along with molecule diameter and differences in lipophilicity, molecular weight may influence membrane permeability, thus causing differences in distribution (Wittsiepe et al. 2007).

Although studies show an association between individual characteristics and the pharmacokinetics of dioxins, furans, and PCBs in the human body, there is no standard method for determining a chemical’s half-life as a function of these factors. Most half-life studies for dioxins, furans, and PCBs follow accidental or occupational exposures, and no single study exists covering the life span of people with varying physical characteristics. Despite summaries of pharmacokinetic data of dioxins, furans (Ogura 2004), and PCBs (Lotti 2003), estimations of exposure and body burden have been hindered by the absence of a half-life range and value for each congener.

In this study we provide congener-specific reference half-life values for adults and infants and a method of half-life estimation based on individual characteristics. Based on a literature search, we defined values that approximate the half-life for 29 selected PCDD, PCDF, and PCB congeners in infants and adults. We examined the relationships between half-life and individual characteristics, and present an equation that uses the chosen reference values to predict half-lives based on these individual characteristics.

## Materials and Methods

We conducted an extensive literature search for human half-life or decay values for the 29 congeners of dioxins, furans, and dioxin-like PCBs included in the World Health Organization 2005 toxic equivalency factor (TEF) scheme (Van den Berg et al. 2006). Measured or modeled half-life values for each congener and the age of the subject or mean age of the cohort were recorded from > 30 studies (Tables 1–4).

We selected a subset of data based on the following criteria: blood serum concentrations < 700 ppt total toxic equivalents (TEQs) at the time of sampling, adult subjects, and measure ments not reported as inaccurate in later studies. We retained half-life values that were calculated assuming steady-state conditions if they were < 25 years, because this assumption is inappropriate for more persistent substances with significantly higher historical levels. The mean and range of half-lives were calculated for the retained subset to establish a representative set of half-lives for each congener in a moderately exposed adult.

We selected the adult reference values to represent a 40- to 50-year-old with blood dioxin concentrations in the range where fat drives the rate of elimination. We preferentially chose sources that provided consistent data across congeners and that were within the range of all measured data. Infant reference values were chosen to represent an individual < 2 years of age. When infant data were not available, we multiplied the adult reference value for the congener by the ratio of the length of the adult half-life over the infant half-life for TCDD.

We examined half-life variation as a function of individual characteristics. When the mean age of the cohort was not explicitly provided, we estimated the mean age at the midpoint of sampling. When percent body fat or total body fat data were not available, we converted the mean age-specific BMI reported in the National Health and Nutrition Examination Survey (NHANES) 2003–2004 study [Centers for Disease Control and Prevention (CDC) 2006] to percent body fat. For adults, we used the approach proposed by Deurenberg et al. (1991):


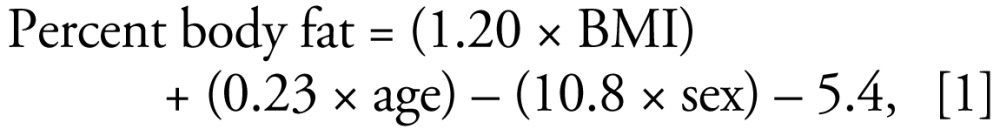


where sex corresponds to females = 0, and males = 1. We used this approach in adults because, unlike the method developed by Knapik et al. (1983) that is used by Flesch-Janys et al. (1996) and the Ranch Hand cohort analysis (Michalek et al 1996; Wolfe et al. 1994), it takes into account both age and sex. Studies have shown that if age is not included in the conversion from BMI to percent body fat, it may seriously underestimate percent body fat in older people (Deurenberg et al. 1991; Hattis et al. 2003).

In children (0–19 years of age), we used a series of age-based equations presented by Hattis et al. (2003) to predict percent body fat for each age in months. Total body fat was estimated by multiplying the average weight reported in the NHANES data for a given age and sex by the calculated percent body fat (CDC 2006).

Based on the apparent relationships between half-life and these parameters, we propose a procedure of half-life estimation that is a function of age, percent body fat, smoking status, and breast-feeding.

## Results and Discussion

### Review of reported half-life values

A comprehensive report of half-life values for dioxins, furans, and PCBs is presented in Tables 1–4. Studies that are listed more than once are those that report multiple half-life values, generally corresponding to measurements on different individuals. Of the studies examined, one-third are limited to TCDD: five of these report on the Ranch Hand cohort (Michalek et al. 1996, 2002; Michalek and Tripathi 1999; Pirkle et al. 1989; Wolfe et al. 1994), three with kinetic data based on the incident in Seveso, Italy (Kerger et al. 2006; Michalek et al. 2002; Needham et al. 1997), one on a poisoning incident in Austria (Geusau et al. 2002), and two based on an adult male volunteer (Poiger and Schlatter 1986; Schlatter 1991). Sixteen different measurements are based on the Yu-Cheng and Yusho cohorts (Chen et al. 1982; Kashimoto et al. 1983; Leung et al. 2005, 2007; Ryan and Masuda 1989, 1991; Ryan et al. 1993; Shirai and Kissel 1996). Six studies report models or measurements based on occupational exposures (Brown et al. 1989; Flesch-Janys et al. 1996; Rohde et al. 1999; Schecter et al. 1990; Van der Molen et al. 2000; Wolff et al. 1992). Five studies have information only on infants and children (Gorski et al. 1984; Kerger et al. 2007a, 2007b; Kreuzer et al. 1997; Leung et al. 2006; Wolff and Schecter 1991), and two data sets are based on general populations (Ogura 2004). The average number of values for dioxins and furans is 10, and among the PCBs the average is 4. No half-life data were available for 1,2,3,7,8,9-HxCDF.

**Figure 1:**
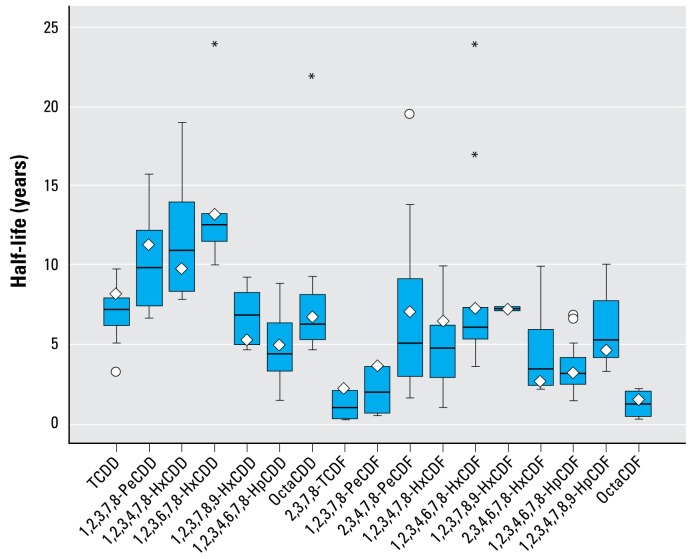
Range of half-life values (in years) for dioxins and furans based on a subset of values from the literature. Bars represent 25th, 50th, and 75th percentiles, and whiskers indicate the range. Diamonds indicate the reference values within this range, circles indicate outliers, and asterisks indicate extreme cases.

**Figure 2:**
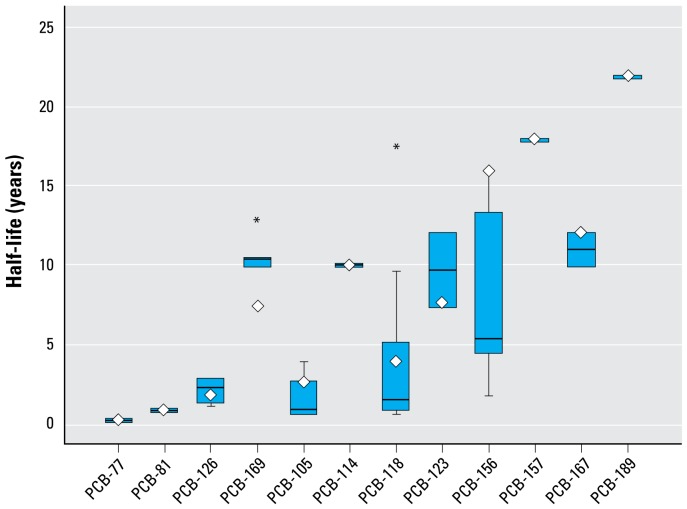
Range of half-life values (in years) for PCBs based on a subset of values from the literature. Bars represent 25th, 50th, and 75th percentiles, and whiskers indicate the range. Diamonds indicate the reference values within this range, circles indicate outliers, and asterisks indicate extreme cases.

The ranges of the subsets of reported values for adults are shown in Figure 1 (dioxins and furans) and Figure 2 (PCBs), and the values are shown in Tables 1–3. The comparison of reported half-life values reveals large variation across congeners. For example, the mean half-lives of octachlorinated dibenzo-furan (octaCDF), tetrachlorinated dibenzofuran (TCDF), and 1,2,3,7,8-PeCDF are all < 3 years, whereas the mean half-lives for some of the HxCDD congeners are more than a decade. The half-lives in the PCBs range from only a few months (PCB-77) to a few decades (PCB-157), and one study reported a > 100-fold range in metabolic clearance rates between PCB congeners (Brown and Lawton 2001).

Within each congener, half-life values reported from the literature vary substantially, typically by a factor of 2–3, but up to a factor of 35 within the subset. This variation may be a result of different exposure concentrations or scenarios, differences in the demographics of the considered cohort, or differences in the pharmacokinetic model used in half-life calculation. Several studies reported on a single person or had very small sample sizes, resulting in unstable mean values. For example, the 15.7-year half-life reported by Flesch-Janys et al. (1996) for 1,2,3,7,8-PeCDD became 11 years when they excluded one worker close to background. Some of the variability in reported half-life values can be explained through differences in physiologic processes among individuals and different congener properties. However, very short half-lives (i.e., < 1 year) are unlikely for the most frequently found congeners because of the high exposures required to sustain measurable body burdens, and very long half-lives (> 10 years) may be artifacts of ongoing exposures (Shirai and Kissel 1996).

Most cohorts consist of adult males exposed to high concentrations, although measurements were sometimes carried out years after exposure. Half-life measurements for persons at or near background levels, including those with no history of substantial exposure or those who have returned to background levels after significant exposure, may be confounded by the effect of probable continuous exposure to background levels of dioxins. Half-life measurements and the influence of other factors (e.g., smoking, body fat) may be better evaluated when sampled from persons with higher accidental exposures, if concentration-dependent effects can be clearly accounted for.

Most of the studies report concentrations normalized by gram of lipid and assume a conserved equilibrium between dioxins and lipids across the body. The suitability of this measurement to calculate the overall body burden depends on the distribution of the congener into adipose tissue. Although different congeners partition differently into different organs (Iida et al. 2007; Kitamura et al. 2001), a correlation between levels in the blood and levels in adipose tissue is supported (Iida et al. 1999).

### Variation in half-life as a function of age

**Figure 3:**
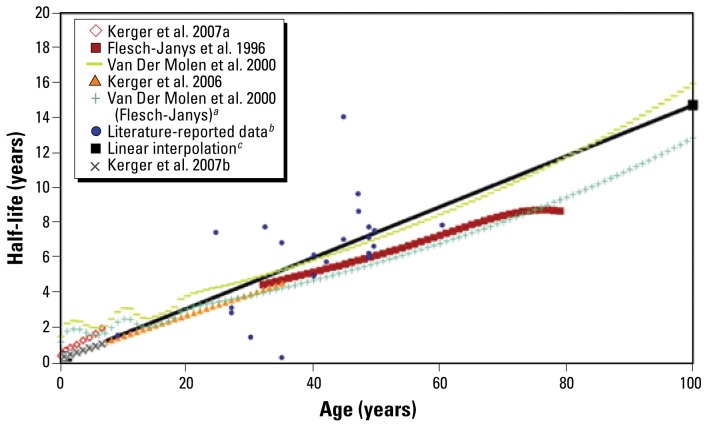
Half-life of TCDD as a function of age. ***a***Application of the model presented by Van der Molen et al. (2000) to the Flesch-Janys et al. (1996) data as done by Ogura (2004). ***b***Values from the current literature presented in Table 1. ***c***Linear interpolation between the infant and adult reference half-lives (slope and intercept given in Table 5).

We observed a positive association between age and half-life (Figure 3). Although this may indicate a direct relationship between age and half-life, it also incorporates the effect of other parameters, such as age-related changes in percent body fat. We included the influence of body fat, using BMI as a surrogate, in the displayed regressions, which use the mean age-specific BMI reported for the 2003–2004 NHANES study (CDC 2006).

**Figure 4:**
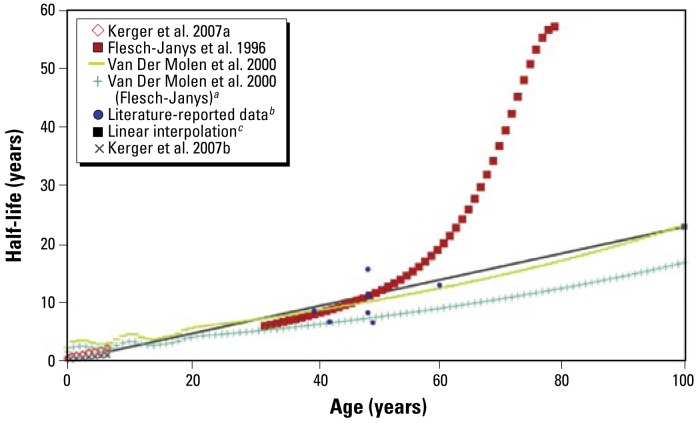
Half-life of 1,2,3,7,8-PeCDD as a function of age. Application of the equation proposed by Flesch-Janys et al. (1996) for ages > 60 years may be problematic because very small variations in the elimination rate could lead to substantial divergence in half-life length. ***a***Application of the model presented by Van der Molen et al. (2000) to the Flesch-Janys et al. (1996) data as done by Ogura (2004). ***b***Values from the current literature presented in Table 1. ***c***Linear interpolation between the infant and adult reference half-lives (slope and intercept given in Table 5).

**Figure 5:**
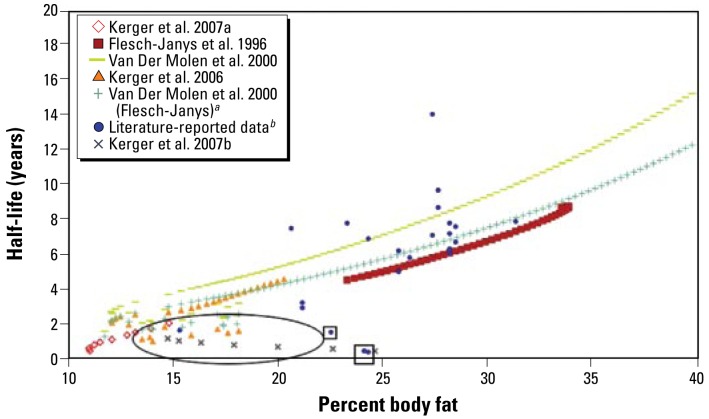
TCDD half-life as a function of percent body fat. The oval indicates the area where the relationship of increased half-life with increased body fat does not hold; these values represent young subjects. Literature-reported data enclosed in squares indicate subjects whose half-lives were measured when they had serum concentrations that were well above the level of increased induction of degradation enzymes. ***a***Application of the model presented by Van der Molen et al. (2000) to the Flesch-Janys et al. (1996) data as done by Ogura (2004). ***b***Values from the current literature presented in Table 1.

**Figure 6:**
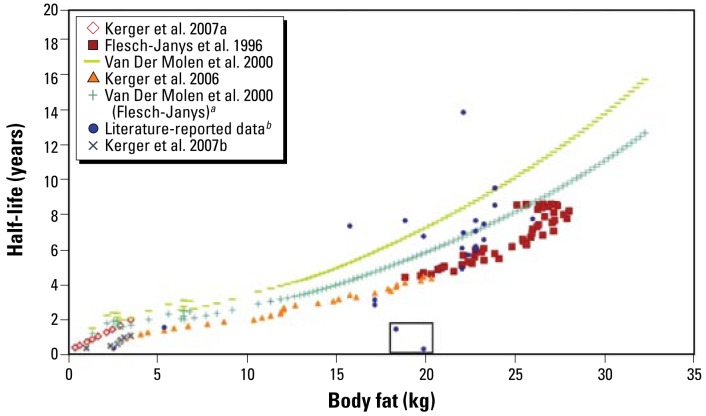
TCDD half-life as a function of total body fat. The two points shown in the square represent subjects whose half-lives were measured when they had serum concentrations well above the level of increased induction of degradation enzymes. ***a***Application of the model presented by Van der Molen et al. (2000) to the Flesch-Janys et al. (1996) data as done by Ogura (2004). ***b***Values from the current literature presented in Table 1.

The points representing literature-reported data in Figures 3–6 are generally averages of a range of ages and a range of half-life values. These ranges, where available, are presented in Tables 1–3. Application of the model proposed by Van der Molen et al. (2000) results in nonlinear variations at low ages. These variations are linked to modeled variations in body fat during adolescence, but have not been confirmed by experimental data.

The Kerger et al. (2006) data correspond to children with concentrations < 700 ppt and support the hypothesis of a close to linear increase in half-life between ages 0–35 years. The slopes calculated with this method were similar to slopes for adults calculated with the method provided by Flesch-Janys et al. (1996), spanning adults 30–80 years of age. However, the equation proposed by Flesch-Janys et al. (1996) may be problematic for ages > 60 years because very small variations in the elimination rate could lead to substantial divergence in half-life length, as observed in the case of 1,2,3,7,8-PeCDD (Figure 4).

Overall, we observed a nearly linear association between half-life and age, which is most likely linked to the combined effects of growth-caused dilution at young ages and an increase in body fat at older ages. However, this association does not account for inter-individual variation at each age.

### Variation of half-life with body fat

Percent body fat is a good predictor of half-lives in adults, as shown for TCDD in Figure 5. This method is inappropriate for infants and children (identified by oval in figure) because of drastic changes in percent body fat and short half-lives.

The discrepancy between percent body fat and half-lives observed at young ages suggests the use of absolute body fat mass to account for the effect of fat over the entire age range (Figure 6). We obtained total body fat by multiplying calculated percent body fat by age-specific NHANES weight averages (CDC 2006). Further data collection is needed to confirm the validity of the relationship between body fat mass and half-life.

### Reference half-life values

We preferentially used the regression method used by Flesch-Janys et al. (1996) for adult reference half-life values because it covers multiple congeners in a consistent way and incorporates information for percent body fat, sex, and smoking status, and because the resulting values are within the range of the other values in the literature. In the case of TCDD, we used the single median value given by Flesch-Janys et al. (1996) as the reference value, because of its consistency with other reported data. For dioxin and furan congeners not reported by Flesch-Janys et al. (1996), we used the model proposed by Van der Molen et al. (2000) to determine a reference half-life, using the median age (48.7 years) and percent body fat (21.9%) from Flesch-Janys et al. (1996). For 1,2,3,7,8,9-HxCDF, which had no available half-life data, we used the reference half-life for 1,2,3,6,7,8-HxCDF.

We based reference half-lives of PCB-77 and PCB-81 on measurements from samples of adipose tissue, whereas we determined reference half-lives for the 10 remaining PCB congeners based on measurements of blood (Ogura 2004). These values correspond to half-lives observed in the general Japanese population, assuming steady-state conditions. Because of the large decrease in dioxin, furan, and PCB concentrations in the environment in the last 30 years, the steady-state assumption is only appropriate for congeners with half-lives that are significantly shorter than the time elapsed from the peak in environmental concentrations; the half-lives of more persistent congeners could be overestimated.

We based reference half-life values for infants on congener-specific values reported by Leung et al. (2006) where available. These values are modeled estimates based on earlier reported concentration data for PCDD and PCDF congeners in breast-fed infants (Abraham et al. 1996, 1998). These reference values are based on existing data, and better numbers may be available with the generation of new data. In some cases, it may be appropriate to use the median values, also provided in Tables 5 and 6.

### Methods for individual half-life calculation

Based on the relationships discussed above, we propose two methods to predict individualized apparent half-lives of dioxins, furans, and PCBs over a lifetime. We specifically focused on half-lives resulting from moderate levels of exposure, comparable to those resulting from the general exposure of the U.S. population. The use of a simple multilinear regression model to predict half-life as a function of age and BMI or body fat is problematic because data for age and BMI coefficients are lacking for several congeners, and as previously discussed, percent body fat is not a good predictor of half-lives at young ages.

To overcome these limitations, the first method that we propose is a linear relationship of half-lives with age. We found the slope (β_age_) and the intercept [β_0(age)_] coefficients by using a linear interpolation between the infant and adult reference half-lives (shown in Tables 5 and 6). We accounted for interindividual variation in body composition and smoking habits with two multiplicative factors (Equation 2). The observed linear influence, supported by modeled results (Emond et al. 2006), of the percent body fat at age = *i* was incorporated in the calculation by multiplying the original equation by the ratio of the individual percent body fat (pbf*_i_*) to the reference percent body fat for that age [pbf_ref(age_*_i_*_)_]. We determined the reference percent body fat by converting the age-specific BMI values from the NHANES data to percent body fat using the method proposed by Deurenberg et al. (1991) and presented above. Similarly, we introduced the effect of smoking through a unitless multiplicative smoking factor (SF*_i_*). The ratios of the decay rate of smokers to nonsmokers in Flesch-Janys et al. (1996) were used when available, ranging from 0.5 to 0.7, corresponding to a 50% to 30% reduction in half-life (Tables 5 and 6); when not available, we used the geometric mean of all available smoking factors, corresponding to a 35% reduction in half-life. We accounted for differences between sexes indirectly by the different percent body fat values for males and females at each age. The predicted half-life (years) for an individual *i* as a function of age, smoking status, and percent body fat *i* was calculated using the empirical model formalized by Equation2:


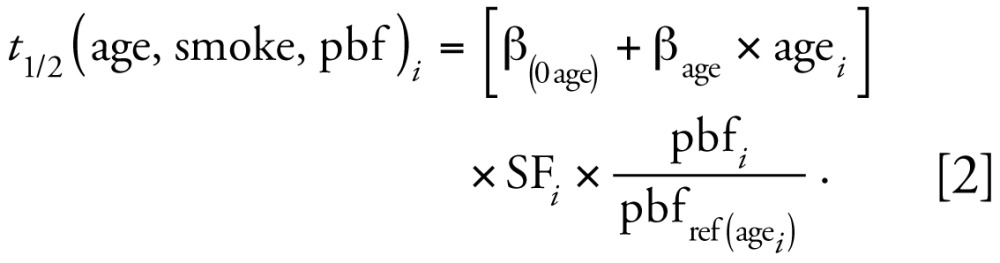


This equation estimates adult half-lives that are comparable to those obtained with the approach proposed by Flesch-Janys et al. (1996) (see Supplemental Material, Figure 1; available online at http://www.ehponline.org/members/2008/11781/suppl.pdf), while extending its applicability to children and to adults > 60 years of age.

A mathematical equation describing the additional rate of elimination due to breast-feeding (Equation 3) is based on the observed effect of breast-feeding in a cohort of German women (Wittsiepe et al. 2007). According to that study, a breast-feeding woman expels an estimated 8.76 kg fat per year through lactation [*q_f_* (kg/day), 0.8 kg milk/day of average 3% lipid], and partition coefficients between blood lipid and milk fat for each congener (*K*_BM_, unitless) range from 0.5 and 4.3 (Tables 5 and 6) (Wittsiepe et al. 2007). Δ*t*_bfed_ (unitless) represents the fraction of the considered year during which the woman was actively breast-feeding, and pbf*_i_* (%) and BW*_i_* (kg) are the woman’s percent body fat and body weight, respectively.


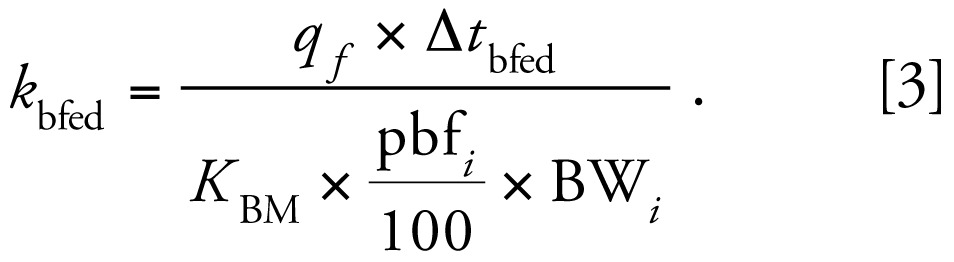


Assuming no interaction between breast-feeding and the other half-life determinants, the overall predicted apparent half-life for a woman who is actively breast-feeding is obtained by adding the effect of elimination through breast-feeding to other age-adjusted, smoking-adjusted, and body-fat–adjusted processes.


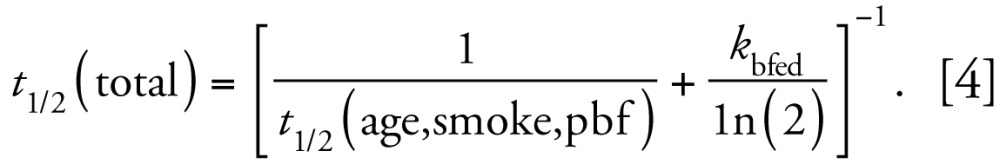


This method predicts a half-life of 4.3 years for TCDD in a 30-year-old, non-smoking woman with 30% body fat if she did not breast-feed that year, and a half-life of 1.8 years if she breast-fed for 6 months.

The alternative proposed strategy to model congener-specific half-lives is based on an observed apparently linear relationship (Figure 6) with absolute body fat, formalized as follows, using the same correction for smoking status as in Equation2 :


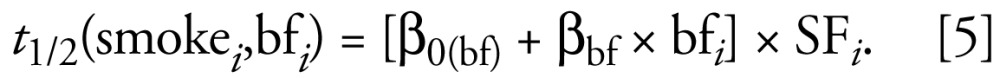


There is insufficient data to test this equation, so this approach requires further data collection and validation.

## Conclusion

Reported half-lives of dioxin and dioxin-like congeners in humans vary widely between and within different dioxin, furan, and PCB congeners. Age, a measure of body fat, smoking habits, and breast-feeding status are strong determinants of the elimination rates observed in humans. The present study integrates these critical factors into an empirical model that predicts the half-lives of the 29 World Health Organization TEF scheme congeners over a human life span. We support a method of half-life estimation that is a function of age. We found a nearly linear relationship between half-life and body fat, but further study and new data are required to evaluate the validity of any estimation methods based on this approach.

Pharmacokinetic information is scarce for many PCB congeners, and many existing studies report on PCB mixtures rather than individual congeners. Further, many of the existing data sets do not take into account the effect of ongoing exposures to background levels. The half-life range and reference values may be refined as more congener-specific data becomes available. Pharmacokinetic studies across multiple congeners, which take into consideration demographic factors, are necessary to determine accurate elimination rates. Further study into the causes of interindividual and intra-individual elimination rate variability, such as the effect of genetic polymorphisms and sensitivity to known factors, would refine half-life estimation accuracy.

The equations described here represent a simple and relatively consistent approach that can be used to determine individual apparent half-lives for numerous dioxin, furan, and PCB congeners. Median and reference values are representative of average behavior rather than extremes. These values cannot be used for highly exposed persons, for whom high TEQ will induce higher elimination. However, the proposed method of half-life prediction can be used to relate past and present intake to serum concentrations and is useful to understand the effect of demographic characteristics on serum concentrations.

## Figures and Tables

**Table 1:** Congener-specific half-lives [median (range) or parametric estimate] for dioxins from the literature.

Study	TCDD	1,2,3,7,8-PeCDD	1,2,3,4,7,8-HxCDD	1,2,3,6,7,8-HxCDD	1,2,3,7,8,9-HxCDD	1,2,3,4,6,7,8-HpCDD	OctaCDD
Flesch-Janys et al. 1996 [median (range)]	7.2 (2.5–∞)	15.7 (3.6–∞)	8.4 (1.4–∞)	13.1 (2.9–∞)	4.9 (2.0–∞)	3.7 (1.6–16.1)	6.7 (1.8–∞)
Flesch-Janys et al. 1996 (parametric estimate)	6.1	11.2	9.8	13.1	5.1	4.9	6.7
Rohde et al. 1999	9.2 (5.8–15.4)	13.9 (9.9–23.1)	13.9 (7.7–19.8)	11.6 (4.3–23.1)	7.7 (5–9.2)	4.3 (2.9–5.8)	8.7 (5.8–11.6)
Geusau et al. 2002 (patient 1)	1.5^a^						
Geusau et al. 2002 (patient 2)	2.9^a^						
Gorski et al. 1984				3.5^a^		3.2^a^	5.7^a^
Leung et al. 2006 (infant 1)	0.43^a^	0.36^a^		0.44^a^		0.36^a^	0.5^a^
Leung et al. 2006 (infant 2)	0.36^a^	0.28^a^		0.33^a^		0.28^a^	0.42^a^
Poiger and Schlatter 1986	5.8^a^						
Schlatter 1991	9.7						
Pirkle et al. 1989	7.1 (5.8–9.6)						
Wolfe et al. 1994	11.3 (10.0–14.1)	a					
Michalek et al. 1996	8.7 (8.0–9.5)						
Michalek and Tripathi 1999	7.6 (7.0–8.2)						
Michalek et al. 2002	7.5 (4.5–∞)						
Kerger et al. 2006 (age < 18 years_)_	1.6^a^						
Kerger et al. 2006 (age > 18 years)	3.2^a^						
Michalek et al. 2002 (first 0.27 years)	0.34 (0.16–∞)^a^						
Michalek et al. 2002 (3–16.35 years)	6.9 (4.15–∞)						
Needham et al. 1994	7.8						
Kreuzer et al. 1997 (infant)	0.4^a^						
Kreuzer et al. 1997 (adult)	5^a^						
Ogura 2004 (blood)		6.7 (4.9–9.6)		42 (29–60)^a^		5.8 (4.0–8.3)	22 (18–26)
Ogura 2004 (adipose)	6.7 (3.3–14)	6.6 (3.6–12)		24 (12–50)	9.2 (3.2–27)	1.4 (0.7–3.0)	5 (1.8–14)
Liem and Theelen 1997^b^	6.2	8.6	19	70^a^	8.5	6.6	5.6
Liem and Theelen 1997; Ogura 2004^c^	7.8	11	12	12	6.8	8.8	5.7
Flesch-Janys et al. 1996; Ogura 2004^c^	6.3	8.3	7.8	10	4.6	3.2	4.6

∞ (Infinity) indicates that at least one person had an increase in serum concentrations between measurements.

a Values that fit exclusionary criteria for the subset.

b As reported in Ogura (2004).

c Application of model in Ogura (2004).

**Table 2:** Congener-specific half-lives for furans from the literature.

Study	2,3,7,8- TCDF	1,2,3,7,8- PeCDF	2,3,4,7,8- PeCDF	1,2,3,4,7,8- HxCDF	1,2,3,6,7,8- HxCDF	2,3,4,6,7,8- HxCDF	1,2,3,4,6,7,8- HpCDF	1,2,3,4,7,8,9- HpCDF	OctaCDF
Flesch-Janys et al. 1996 [median (range)]			19.6 (12.6–31.5)	6.2 (1.9–∞)	6 (2.1–∞)	5.8 (3.1–19.8)	3 (2.1–∞)	3.2 (2.1–∞)	
Flesch-Janys et al. 1996				6.4	7.2		3.1		
Rohde et al. (1999) [mean (range)]			13.9 (4.6–23.1)	8.7 (4.1–17.3)	5.8 (3.6–9.2)	9.9 (8.7–12.6)	3.9 (2.5–4.6)		
Gorski et al. 1984							1.7^a^		1.8^a^
Leung et al. 2006 (infant 1)			0.23^a^						
Leung et al. 2006 (infant 2)			0.3^a^						
Schecter et al. 1990 (adipose)			4.7	2.9	3.5		6.5		
Schecter et al. 1990 (blood)			7.2	4.4	4.3		4.1		
Schecter et al. 1990 (combined)			4.5	4	4.9		6.8		
Masuda et al. 1995			3.1	3.3			2.4		
Ryan and Masuda 1989^b^		1.7 (1.3–2.9)	2.4 (2.1–5.1)			2.4 (1.6–6.1)			
Ryan et al. 1993 (patient 1)			1.9^a^	2.1^a^			2.9^a^		
Ryan et al. 1993 (patient 2)			2.3	2.9			2		
Ryan et al. 1993 (patient 3)			2.2	2.7			2.1		
Iida et al. 1995			9.1	8.6					
Masuda 2001, 0.6–15.6 years after onset [median (range)]			2.9 (2.7–3.6)	3.5 (2.7–3.6)			2.5 (2.2–2.6)		
Kashimoto et al. 1983			1.5	1.5					
Leung et al. 2005 (> 3 ppb)		1.1^a^	2.3^a^			1.5^a^			
Leung et al. 2005 (< 3 ppb)		7.5	5.9			3.6			
Leung et al. 2007 (> 3 ppb)		1.1	2.3			1.5			
Leung et al. 2007 (< 3 ppb)		7.2	5.7			3.5			
Masuda 2001, 14.0–29.1 years after onset [median (range)]			7.7 (5.2–14.3)	5.1 (3.9–6.9)			3.5 (2.6–6.6)		
Masuda et al. 1995			8.9	5.4			3.9		
Ryan et al. 1993 [median (range)]			9.6 (5.7–36)	7.8 (4.3–54)					
Ogura 2004 (blood) [mean (95% CI)]			4.9 (3.3–7.1)	9.9 (6.6–15)	17 (11–26)		4.8 (3.2–7.2)		
Ogura 2004 (adipose) [mean (95% CI)]	0.2 (0.1–0.4)	0.4 (0.2–1.0)	5 (2.7–9.1)	3.7 (1.3–10)	5.8 (1.4–25)	2.1 (0.8–5.8)	1.4 (0.5–3.8)		2.1 (0.7–6.2)
Liem and Theelen 1997^c^	0.4	0.9	9.9	5.7	6.2	2.4	2.6		0.2
Liem and Theelen 1997; Ogura 2004^d^	1.4	2.9	10	7.7	24	3.6	5	10	0.7
Flesch-Janys et al. 1996^d^,^e^	2.4	3.9	7.8	5.6	7.1	3.1	2.8	5.2	1.6

Values shown are parametric estimates except where indicated. ∞ (Infinity) indicates that at least one person had an increase in serum concentrations between measurements.

a Values that fit exclusionary criteria for the subset.

b Value not defined.

c As reported in Ogura (2004).

d Application of model in Ogura (2004).

e Also reported a parametric estimate of 7.1 for 1,2,3,7,8,9-HxCDF.

**Table 3:** Congener-specific half-lives for PCBs from the literature.

Study	PCB-77	PCB-81	PCB-126	PCB-169	PCB-105	PCB-114	PCB-118	PCB-123	PCB-156	PCB-157	PCB-167	PCB-189
Masuda et al. 1995							1.7		4.9			
Shirai and Kissel 1996				10.4			1.1		1.62			
Ryan et al. 1993 (patient 1)^a^							1.1^b^		3.3^b^			
Ryan et al. 1993 (patient 2)^a^							1.2		5.4			
Ryan et al. 1993 (patient 3)^a^							1.3		4			
Chen et al. 1982					0.56		0.82					
Shirai and Kissel 1996^c^					0.58		0.83		∞^b^			
Shirai and Kissel 1996^d^					0.51		0.77		∞^b^			
Masuda 2001, 0.6–15.6 years after onset [median (range)]							1.6 (1.5–1.9)		5.3 (3.8–5.6)			
Masuda 2001, 14.0–29.1 years after onset [median (range)]							17.6 (6.9–33.7)		13.2 (8.5–21.5)			
Ryan and Masuda 1991,												
Masuda et al. 1995							17.6		13.4			
Ryan et al. 1993^e^				10.4								
Brown et al. 1989					3.9		5.8					
Brown and Lawton 2001	5.02		11		7.0	31.7^b^	10.8	15.3	100^b^	20^b^	35^b^	166.7^b^
Buhler et al. 1988							0.5^b^					
Wolff and Schecter 1991										4.6^b^		
Wolff et al. 1992 [mean (range)]					∞^b^		9.6 (7.4–23)					
Ogura 2004 (blood) [mean (95% CI)]			1.6 (1.2–2.1)	7.3 (5.2–10.4)	2.4 (1.7–3.3)	10 (7.4–14.2)	3.8 (2.8–5.3)	7.4 (5.3–10)	16 (11–23)	18 (13–26)	12 (8.7–17)	22 (16–32)
Ogura 2004) (adipose) [mean (95% CI)]	0.1	0.7 (0.4–1.2)	2.7 (1.6–4.5)	13 (8.8–19)	2.7 (1.5–4.8)	25 (16–40)^b^	4.2 (2.3–7.5)	12 (5.8–25)	38 (23–63)^b^	27 (16–44)^b^	10 (5.2–19)	41 (24–69)^b^
Liem and Theelen 1997	0.1		2.7									

Values shown are parametric estimates except where indicated. ∞ (Infinity) indicates that at least one person had an increase in serum concentrations between measurements.

a Yu-Cheng.

b Values that fit exclusionary criteria for the subset.

c First and second samples from Chen al. (1982).

d First and third samples from Chen al. (1982).

e Yusho.

**Table 4:** Characteristics and study information for studies with congener-specific half-life data.

Study	Age (years)	No.	Time from exposure (years)	Time of follow-up (years)	Cohort
Flesch-Janys et al. 1996	32–79 (mean = 48.7)	43	0–37 (mean 5.4)	1–9 (mean 5.6)	Occupational^a^
Rohde et al. 1999	41–73	6	—	4–6	Occupational^b^
Geusau et al. 2002	27, 30	2	0	3	Poisoned Austrian women
Gorski et al. 1984	Child	1	—	2.5	Child (wood in home)^c^
Leung et al. 2006	Infant	2	0	1	Breast-fed infants
Schecter et al. 1990	Late 50s to early 60s	1	2	3	Occupational
Poiger and Schlatter 1986	42	1	0	< 1	Adult male volunteer
Schlatter 1991	47	1	—	6	Adult male volunteer^d^
Pirkle et al. 1989	—	36	> 10	5	Ranch Hand
Wolfe et al. 1994	31.8–66	337	> 10	5	Ranch Hand^e^
Michalek et al. 1996	31.8–66	213	14.8	10.3	Ranch Hand^e^
Michalek et al. 1999	31.8–66	97	> 9.3	15	Ranch Hand^e^
Michalek et al. 2002	18–38	97	9–33	15	Ranch Hand^f^
Kerger et al. 2006	0.5–16.6	45	0	17	Seveso
Kerger et al. 2006	> 18	45	0	17	Seveso
Michalek et al. 2002	16–71	35	0	0.27	Seveso
Michalek et al. 2002	16–71	54	3	13.35	Seveso
Needham et al. 1994	—	27	—	—	Seveso^c^
Kreuzer et al. 1997	< 1	20	0	< 1	Infants
Kreuzer et al. 1997	40	—	—	—	Model based on infants^a^
Masuda et al. 1995	25	3	0.6	15	Yu-Cheng^c^,^g^
Ryan and Masuda 1989	—	2–4		—	Yu-Cheng^h^
Shirai and Kissel 1996	17–69	19	1–14	8–9	Yu-Cheng and Yusho^i^
Ryan et al. 1993	17, 25, 33	3	1–10	9	Yu-Cheng (individual)
Iida et al. 1995	—	7	14	1	Yu-Cheng and Yusho^b^,^j^
Chen et al. 1982	—	17	0	1	Yu-Cheng
Shirai and Kissel 1996	—	20–24	< 1	0.7–4.7	Yu-Cheng^i^
Masuda 2001	17–33	3	1	15	Yu-Cheng
Kashimoto et al. 1983	—	30	< 1	1–2	Yu-Cheng^k^
Leung et al. 2005	18–80	8	1–14	15	Yu-Cheng and Yusho
Leung et al. 2007	18–80	8	1–14	15–19	Yu-Cheng and Yusho
Masuda 2001	31–51	5	14	16	Yusho
Masuda et al. 1995	—	5	—	—	Yusho^c^,^g^
Ryan et al. 1993	33–69	16	14–22	8	Yusho (five individuals)
Brown et al. 1989	—	39	1–26	7.7	Occupational
Brown and Lawton 2001	—	1–10	1–6	11	Occupational^l^
Buhler et al. 1988	50	1	< 1	< 1	Male volunteer
Wolff and Schecter 1991	2–6	4,5	—	—	Children, contaminated material^m^,^n^
Wolff et al. 1992	45	18–165	< 1	3.83	Occupational
Ogura 2004 (blood)	20–65	253	—	—	General Japanese population
Ogura 2004 (adipose)	40–59	10	—	—	General Japanese population
Liem and Theelen 1997	—	—	—	—	General Dutch population^c^
Liem and Theelen 1997; Ogura 2004	—	—	—	—	General Dutch population^a^,^o^
Flesch-Janys et al. 1996; Ogura 2004	48.7	—	—	—	Occupational^a^,^o^

— , not available.

a Modeled value.

b Fecal clearance only.

c Data accessed from Ogura (2004).

d Data accessed from Flesch-Janys et al. (1996).

e Age in 1982.

f Age during tour of duty.

g Also published by Ryan and Masuda (1991).

h Data accessed from U.S. Environmental Protection Agency (2000).

i Application of model presented to data from study in Chen et al. (1982).

j Data accessed from Ryan et al. (1993).

k Data accessed from abstract.

l Reported two metabolic clearance rates, not apparent half-life values; clearance rates were assumed to be additive, and half-lives were calculated as follows: *t*_1/2_ = 1/*k_a_* + 1/*k_b_*.

m Did not account for growth; may be near background.

n Data accessed from Shirai and Kissel (1996).

o Application of kinetic model to data.

**Table 5:** Reference half-lives (in years) and model parameters for Equations 2 and 3 for dioxins and furans.

	Infant half-life	Adult half-life	Median half-life	Reference adult age (years)	Source (adult values)	SF	*K*_BM_	Intercept (**β**_0_)	Slope (**β**_age_)
TCDD	0.4^a^	7.2	6.3	48.7	b	0.739	0.92	0.26	0.15
1,2,3,7,8-PeCDD	0.3^a^	11.2	8.5	48.7	c	0.683	1.21	0.09	0.23
1,2,3,4,7,8-HxCDD	0.5	9.8	10.90	48.7	c	0.509	1.44	0.35	0.20
1,2,3,6,7,8-HxCDD	0.4^a^	13.1	12	48.7	c	0.635	1.32	0.12	0.27
1,2,3,7,8,9-HxCDD	0.3^a^	5.10	6.8	48.7	c	0.665	1.51	0.18	0.10
1,2,3,4,6,7,8-HpCDD	0.3^a^	4.9	3.7	48.7	c	0.525	1.87	0.22	0.10
OctaCDD	0.5^a^	6.7	5.7	48.7	c	0.551	3.3	0.33	0.14
2,3,7,8-TCDF	0.1	2.1	0.9	48.7	d	0.648	1.1	0.08	0.04
1,2,3,7,8-PeCDF	0.2	3.50	1.9	48.7	d	0.648	1.6	0.13	0.07
2,3,4,7,8-PeCDF	0.3^a^	7.0	4.9	48.7	d	0.648	1.15	0.13	0.14
1,2,3,4,7,8-HxCDF	0.4	6.4	4.8	48.7	c	0.692	1.79	0.23	0.13
1,2,3,6,7,8-HxCDF	0.4	7.2	6	48.7	c	0.695	1.91	0.26	0.15
1,2,3,7,8,9-HxCDF	0.4	7.2	[Table-fn tfn33-ehp-117-417]—	40.0	e	0.648	1.39^f^	0.19	0.15
2,3,4,6,7,8-HxCDF	0.2	2.8	3.4	48.7	d	0.648	1.38	0.10	0.06
1,2,3,4,6,7,8-HpCDF	0.2	3.1	3	48.7	c	0.832	2.59	0.11	0.06
1,2,3,4,7,8,9-HpCDF	0.3	4.6	5.2	48.7	d	0.648	4.28	0.17	0.09
OctaCDF	0.1	1.4	1.6	48.7	d	0.648	3.4	0.05	0.03

— , not available. *K*_BM_, blood lipid to milk fat ratio; SF, smoking factor.

a Infant reference values taken from Leung et al. (2006).

b Flesch-Janys et al. (1996), median value.

c Flesch-Janys et al. (1996), regression values.

d Van der Molen et al. (2000).

e No data for this congener (the half-life values were taken to be the same as 1,2,3,6,7,8-*HxCDF*).

f Geometric mean of all *K*_BM_ values.

**Table 6:** Reference half-lives (in years) and model parameters for Equations 2 and 3 for PCBs.

	PCB-77	PCB-81	PCB-126	PCB-169	PCB-105	PCB-114	PCB-118	PCB-123	PCB-156	PCB-157	PCB-167	PCB-189
Infant reference half-life (years)	0.0	0.0	0.1	0.4	0.1	0.5	0.2	0.4	0.9	1.0	0.7	1.2
Adult reference half-life (years)	0.1	0.7	1.6	7.3	2.4	10.0	3.8	7.4	16.0	18.0	12.0	22.0
Median half-life	0.1	0.73	2.7	10.4	2.4	25	1.6	12	5.35	20	12	41
Reference adult age (years)	49.5	49.5	42.5	42.5	42.5	42.5	42.5	42.5	42.5	42.5	42.5	42.5
Source	a	a	b	b	b	b	b	b	b	b	b	b
SF	0.648	0.648	0.648	0.648	0.648	0.648	0.648	0.648	0.648	0.648	0.648	0.648
*K*_BM_	1.39^c^	1.39^c^	0.67	1.24	0.72	0.69	0.87	0.52	1.16	1.26	1.19	1.99
Intercept (β_0_)	0.00	0.03	0.05	0.24	0.08	0.33	0.13	0.24	0.53	0.59	0.39	0.72
Slope (β_age_)	0.00	0.01	0.04	0.17	0.06	0.23	0.09	0.17	0.37	0.42	0.28	0.51

*K*_BM_, blood lipid to milk fat ratio; SF, smoking factor.

a Sources of adult reference values: Ogura (2004) blood data;

b Ogura (2004) adipose tissue data.

c Geometric mean of all *K*_BM_ values.
